# Two Metagenome-Assembled Genome Sequences of Magnetotactic Bacteria in the Order *Magnetococcales*

**DOI:** 10.1128/MRA.00363-20

**Published:** 2020-08-27

**Authors:** Wensi Zhang, Runjia Ji, Jia Liu, Yongxin Pan, Long-Fei Wu, Wei Lin

**Affiliations:** a Key Laboratory of Earth and Planetary Physics, Institute of Geology and Geophysics, Chinese Academy of Sciences, Beijing, China; b Innovation Academy for Earth Science, Chinese Academy of Sciences, Beijing, China; c France-China Joint Laboratory for Evolution and Development of Magnetotactic Multicellular Organisms, Chinese Academy of Sciences, Beijing, China; d College of Earth and Planetary Sciences, University of Chinese Academy of Sciences, Beijing, China; e Aix Marseille University, CNRS, LCB, Marseille, France; University of Southern California

## Abstract

Magnetotactic bacteria represent a valuable model system for the study of microbial biomineralization and magnetotaxis. Here, we report two metagenome-assembled genome sequences of uncultivated magnetotactic bacteria belonging to the order *Magnetococcales*. These genomes contain nearly complete magnetosome gene clusters responsible for magnetosome biomineralization.

## ANNOUNCEMENT

Magnetotactic bacteria (MTB) are a diverse group of microorganisms that swim along the geomagnetic field lines, a behavior known as magnetotaxis or microbial magnetoreception (1). MTB affiliated within the order *Magnetococcales* are often the dominant MTB group in nature (2, 3). This group was previously thought to be an order (4) or a subclass (5) near the base of *Alphaproteobacteria* but was recently reclassified as a novel candidate class (“*Candidatus* Etaproteobacteria” [6, 7] or *Magnetococcia* [8]) within the *Proteobacteria* phylum. Genomes of *Magnetococcales* are required to better understand the phylogenetic position, genomic diversity, and evolutionary history of this MTB lineage.

Here, we report two metagenome-assembled *Magnetococcales* genome sequences isolated from freshwater sediments. Surface sediments were collected from East Lake in Hubei Province, China (30.56°N, 114.41°E). MTB cells were magnetically enriched from 300 ml of sediments using a double-ended open magnetic separation apparatus known as the “MTB trap” (9) through 4 h of collection. DNA was directly amplified from magnetically enriched cells using the Genomiphi V2 DNA amplification kit (GE Healthcare, USA) according to the manufacturer’s protocol and purified with the AxyPrep Mag PCR clean-up kit (Axygen, USA). Libraries were prepared with the Nextera XT DNA library preparation kit (Illumina, USA), following the manufacturer’s instructions. DNA sequencing was performed with an Illumina HiSeq 2500 instrument using the paired-end 125-bp by 125-bp library with a 200-bp insert size (BGI-Wuhan, Wuhan, China). Paired-end reads were filtered and trimmed using SOAPnuke (10) and were assembled using metaSPAdes (11) with the following parameters: --only-assembler -k 31, 41, 51, 61, 71, 81, 91, 101, 111. Assembled scaffolds of ≥2,500 bp were binned separately using MetaBAT v0.26.1 (12) and MyCC (13), and the high-scoring, nonredundant set of bins were dereplicated and selected using DASTool (14). QUAST v4.1 (15) was used to assess the quality of acquired genome sequences, and their completeness and contamination were estimated using CheckM (16) (taxonomy_wf domain *Bacteria*). Coverage information was determined using Bowtie 2 v2.3.4.3 (17) and SAMtools v1.6 (18). Genome sequences were annotated using the Prokaryotic Genome Annotation Pipeline (PGAP) (19). Putative magnetosome genes were checked using NCBI PSI-BLAST (20). Average amino acid identity (AAI) values were calculated using enveomics (21). Unless otherwise specified, default parameters were used for all software.

Two distinct (54% of AAI score) genome sequences, designated DH2bin6 and DH2bin20, have been reconstructed here, both of which contain partial 16S rRNA genes (>900 bp). An analysis of the 16S rRNA gene sequence of DH2bin6 using the online NCBI BLASTn nucleotide collection (nonredunant [nr]/nucleotide) database (https://blast.ncbi.nlm.nih.gov) shows the best hit to an uncultured magnetotactic coccus, MDA-1 (GenBank accession number AB537162; 99.89% identity) (22), while DH2bin20 has a 95.84% BLASTn identity to uncultivated Magnetic coccus CS92 (X81182) (23). Genomes of DH2bin6 and DH2bin20 consist of 80 and 525 scaffolds with average GC contents of 56.76% and 52.96%, respectively (Table 1). Nearly complete magnetosome gene clusters, a group of genes responsible for magnetosome biogenesis and arrangement, have been identified in both genomes, which contain genes homologous to magnetosome genes of *mamD*, *mamH*, *mamI*, *mamE*, *mamK*, *mamF*, *mamL*, *mamM*, *mamN*, *mamO*, *mamP*, *mamA*, *mamQ*, *mamB*, *mamS*, *mamT* and *mmsF*. These two genomes will provide insights into the genome biology and magnetosome biomineralization of MTB within the order *Magnetococcales*.

**TABLE 1:** Genome statistics of *Magnetococcales* isolates DH2bin6 and DH2bin20

Name	Genome accession no.	Genome size (bp)	No. of scaffolds	*N*_50_ (bp)	No. of genes	GC content (%)	Completeness (%)	Contamination (%)	No. of mapped reads	Coverage (×)
DH2bin6	JAANAU000000000	3,658,807	80	88,744	3,116	56.76	98.28	0.00	2,522,452	105
DH2bin20	JAANAV000000000	5,014,406	525	14,837	4,487	52.96	93.97	8.93	933,940	31

### Data availability.

These two genome sequences have been deposited in GenBank under the accession numbers JAANAU000000000 and JAANAV000000000 (BioProject number PRJNA400260). The raw metagenomic read data have been deposited in the NCBI Sequence Read Archive under the accession number SRR11267947.
